# Different gene alterations in patients with non-small-cell lung cancer between the eastern and southern China

**DOI:** 10.1016/j.heliyon.2023.e20171

**Published:** 2023-09-14

**Authors:** Chengdong Liu, Kangbao Li, Yi Sui, Hongmei Liu, Yunzhi Zhang, Yuan Lu, Wei Lu, Yongfeng Chen, Gehui Wang, Suqian Xu, Tianmin Xiang, Yongguang Cai, Kenan Huang

**Affiliations:** a Department of thoracic surgery, Naval Medical Center of PLA, 338 Huaihai Road, Changning District, Shanghai 200052, China; b Department of Geriatrics, Gastroenterology Ward, Guangzhou First People's Hospital, School of Medicine, South China University of Technology, Guangzhou 510180, China; c Singlera Genomics Inc., Shanghai 201318, China; d Medical Oncology Department V, Central Hospital of Guangdong Nongken 524002, China; e Department of Thoracic Surgery, Shanghai Changzheng Hospital, Navy Military Medical University, 415 Fengyang Road, Huangpu District, Shanghai 200003, China; f Department of Thoracic Surgery, The First Affiliated Hospital of Soochow University, Suzhou 215006, China

**Keywords:** Lung cancer, gene mutations, C > T transitions, Region-related

## Abstract

Geographical differences are conspicuous in lung cancer, and the distinct molecular features of lung tumor between Western patients and Asian patients have been demonstrated. However, the etiology of non-small-cell lung cancer (NSCLC) and the distribution of associated molecular aberrations in China have not been fully elucidated. The mutational profiles of 12 lung cancer-related genes were investigated in 85 patients from eastern China and 88 patients from southern China who had been histologically confirmed NSCLC. Overall, 93.6% (162/173) of tumor samples harbored at least one somatic alteration. The most frequently mutated genes were *TP53* (56.1%), *EGFR* (50.3%), and *KRAS* (14.5%). We found that *EGFR* mutated much more frequently (60.0% *vs* 40.9%, *P* = 0.012) and *TP53* mutations had significantly lower incidence (47.1% *vs* 64.8%, *P* = 0.019) in eastern cohort than that in southern cohort. Mutational signature analysis revealed a region-related mutagenesis mechanism characterized by a high prevalence of C to T transitions mainly occurring at CpG dinucleotides in southern patients. This study reveals the difference in the mutational features between NSCLC patients in eastern and southern China. The distinct patterns of gene mutation could provide clues for the mechanism of carcinogenesis of lung cancer, offering opportunities to stratify patients into optimal treatment plans based on genomic profiles.

## Highlights

• NSCLC patients in eastern and southern China exhibited different mutational features.
• In eastern and southern cohorts, the most frequently mutated genes were *EGFR* and *TP53*, respectively.
• In southern patients, a high prevalence of C to T transitions occurred at CpG dinucleotides.
• The distinct gene mutation patterns offer opportunities to stratify patients into optimal treatment plans based on genomic profiles.

## 1 Introduction

Lung cancer is a molecularly heterogeneous disease, and the molecular features of this tumor vary greatly by region. For instance, *EGFR* gene mutations can be observed in approximately 10%–20% in Western non-small-cell lung cancer (NSCLC) patients, but as high as 40%–50% in Asian population [1,2]. Moreover, *KRAS* mutations occur in about 20–30% in Western and 10%–15% in east Asian patients with NSCLC [3,4]. In China, the landscape of driver mutations in NSCLC patients from different regions displayed a region-specific mutational profile. Especially, the prevalence of *EGFR*, *ALK*, *ROS1* and *KRAS* mutation was significantly different in NSCLC patients from Qujing City than that from other regions located in Yunnan Province of China [5]. Therefore, comprehensive understanding of the oncogenic mutations of NSCLC patients and their relationship with the regions is of great importance in unrevealing the genetic etiology.

The region-related difference of lung cancer incidence and mortality across China is significantly ranges widely [6,7]. High rates were prone to be clustered around urban areas like Beijing and Shanghai. Although the genomic profiles of lung cancer in Chinese patients were previously reported [[8], [9], [10]], the regional distribution related to etiology and carcinogenesis have yet to be elucidated clearly.

In this study, we conducted next-generation sequencing (NGS) to detect multiple lung cancer-related genes status in NSCLC patients from an eastern and a southern region from China, compared the identified molecular mutation spectrum with the clinical features, and aimed to elucidate the most geographical variation of lung cancer between the two cohorts.

## 2 Methods

### 2.1 Patient recruitment

A total of 85 patients from eastern China and 88 patients from southern China were included according to standard procedures. Samples were collected during December 2016 and December 2020 in Shanghai Changzheng Hospital for eastern cohort and Central Hospital of Guangdong Nongken for southern cohort, respectively. To clarify demographic differences, patients were also screened according to an additional procedure, i.e., only participants with household registration or permanent residence in the local area were finally included in the analysis. All the patients enrolled had been histologically confirmed NSCLC according to World Health Organization criteria based on hematoxylin and eosin staining and reviewing by experienced pathologists, and without any additional therapy when they were enrolled in our study. All tumor tissues included in the study were ensured to be derived from the patient's primary tumor site.

### 2.2 DNA extraction from FFPE samples

All tissue sections were reviewed by pathologists to ensure tumor cell content exceeded 10%. DNA was extracted from FFPE tissues using the QIAamp DNA FFPE Tissue Kit (Qiagen, Germany) according to the manufacturer's protocol. Concentrations were detected by the Qubit Fluorometer (Thermo scientific, USA). Only DNA samples with most fragments over 500 bp and a minimum of 50 ng were included in subsequent processes.

### 2.3 Next-generation sequencing and data analysis

Probe capture-based targeted NGS was performed on 173 tumor samples by the OncoAim® kit (Singlera) as previously described [11]. In brief, the OncoAim panel consists of all exons of 12 lung cancer-related genes, including *ALK*, *BRAF*, *EGFR*, *ERBB2*, *FGFR1*, *KRAS*, *MET*, *NRAS*, *PIK3CA*, *RET*, *ROS1*, and *TP53*, as well as *ALK*, *ROS1*, and *RET* gene rearrangement/fusion. After DNA concentration detection, 50 ng of FFPE DNA was sheared to approximately 250 bp for library construction. End repair, A-tailing, and adapter ligation were performed before target capture with probes provided in the kit. The library product sequencing was performed on the NextSeq 500 using 150 bp paired-end runs. The median depth coverage was over 1000X with uniformity exceeding 90%. The sequencing data was then processed in accordance with the instructions provided by the OncoAim® kit. Single nucleotide variations (SNVs), short insertions and deletions (InDels), copy number variations (CNVs), and gene rearrangements were detected simultaneously. The sequencing reads in the FASTQ format were aligned to human genome reference sequence (hg19) by Burrows‐Wheeler Aligner. The threshold of variant allele frequency for somatic mutations was 1%. For CNVs, amplifications were characterized as genes with thresholds equal to or greater than 4 copies for amplification and 0 copies for homozygous deletions. Gene rearrangement was called with at least one splitting read and two discordant read‐pairs and conﬁrmed on Integrative Genomics Viewer (IGV).

### 2.4 Statistical analysis

R project (version 4.0.4) was used for statistical analysis. The association of detected mutations with demographic and clinical factors was analyzed by the chi-square test or Fisher's exact test. Statistically significant defined as *P* value < 0.05.

## 3 Results

### 3.1 Baseline characters of included patients

The demographic characteristics of the enrolled patients, including age, gender, smoking history, histopathology, are summarized in Table 1. The median age, gender ratio, smoking history, and histological type of the two cohorts were roughly equal. Lung adenocarcinoma (LUAD) accounted for 91.9% of patients in this study, while lung squamous cell carcinoma (LUSC), large cell neuroendocrine carcinoma, and sarcomatoid carcinoma accounted for 5.8%, 1.7%, and 0.6%, respectively.

**Table 1:** Characteristics of patients with NSCLC from Eastern and Southern regions.

Characteristic	All patients (n = 173)	Region	
		Eastern China (n = 85)	Southern China (n = 88)
Age			
Median (range)	63 (38–86)	62 (38–83)	65 (42–86)
**Gender**			
Male	99 (57.2%)	49 (57.6%)	50 (56.8%)
Female	74 (42.8%)	36 (42.4%)	38 (43.2%)
**Smoking history**			
Yes	75 (43.4%)	40 (47.1%)	35 (39.8%)
No	98 (56.6%)	45 (52.9%)	53 (60.2%)
Histopathology			
Adenocarcinoma	159 (91.9%)	78 (91.8%)	81 (92.0%)
Squamous cell carcinoma	10 (5.8%)	4 (4.7%)	6 (6.8%)
Large cell neuroendocrine carcinoma	3 (1.7%)	2 (2.4%)	1 (1.1%)
Sarcomatoid carcinoma	1 (0.6%)	1 (1.2%)	0 (0%)

### 3.2 Genetic profiling in overall population

**Fig. 1:**
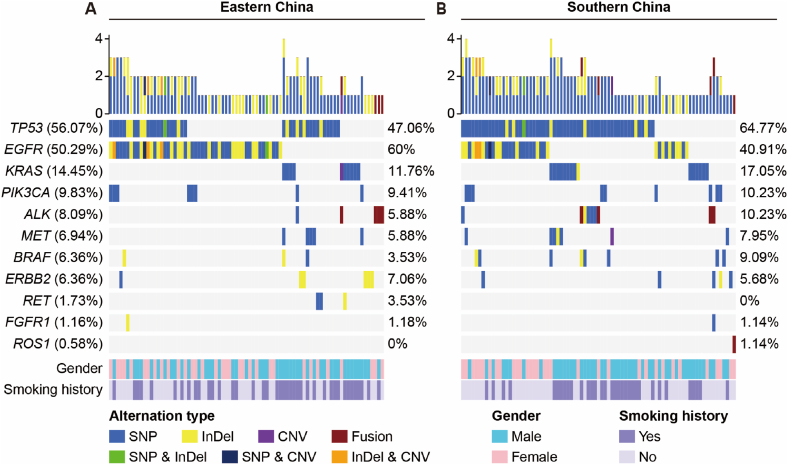
Mutational landscape and clinical characteristics of 162 NSCLC patients with at least one somatic alteration. The X-axis represents 81 patients from eastern China (A) and 81 patients from southern China (B), respectively. The Y-axis represents the mutated genes and the corresponding mutation frequency in all patients (the left column), eastern patients (the middle column) and southern patients (the right column). Clinical characteristics and alteration types are annotated in different colors.

**Fig. 2:**
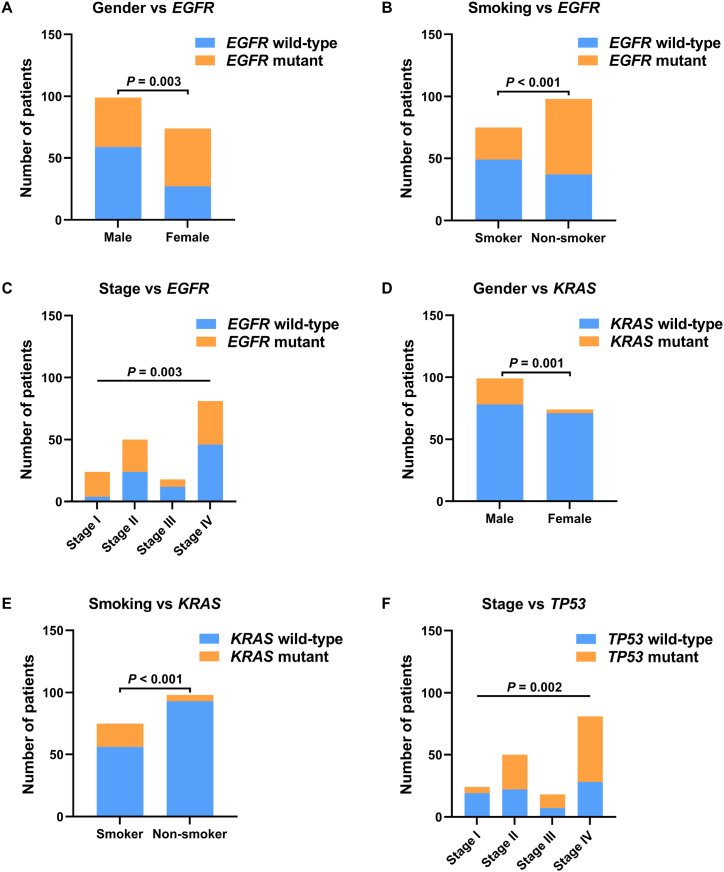
Correlation between clinical characteristics and genetic alterations in NSCLC patients. (**A-C**) Correlation between gender (A), smoking history (B), and stage (C) with *EGFR* mutation in all patients. (**D, E)** Correlation between gender (D) and smoking history (E) with *KRAS* mutation in all patients. (**F**) Correlation between stage with *TP53* mutation in all patients.

Overall, a total of 93.6% (162/173) tumor samples harbored at least one somatic alteration (Fig. 1). SNVs and InDels were identified in 157 samples, gene rearrangements were identified in 9 samples (8 for *ALK* -*EML4*, and 1 for *CD74* -*ROS1*), and CNVs (*EGFR*, *KRAS* and *MET* amplification) were identified in 9 samples. Over 50% of patients in the cohort had mutations in *TP53* or *EGFR* gene, while *KRAS* mutations were present in 14.5% of patients. Mutations in other genes occurred less frequently. We performed chi-square analyses to further identify the correlations between gene mutations and gender, smoking history, or tumor stage (Supplementary Table 1). In brief, *EGFR* mutations were more frequent in female compared to male (63.5% vs 40.4%, *P* = 0.003) (Fig. 2A), in non‐smokers compared to smokers (62.2% vs 34.7%, *P* < 0.001) (Fig. 2B), and in patients with early-stage tumors (83.3% for stage I) compared to middle- or late-stage tumors (52%, 33.3%, and 43.2% for stage II/III/IV, respectively) (Fig. 2C). *KRAS* mutations tended to arise in male rather than female (21.2% vs 4.1%, *P* = 0.001) (Fig. 2D), and in smokers rather than non‐smokers (25.3% vs 5.1%, *P* < 0.001) (Fig. 2E). *TP53* mutations were associated with tumor stage by a lower mutation frequency in stage I (20.8%) compared to stage II/III/IV (56%, 61.1%, and 65.4%, respectively) (Fig. 2F).

### 3.3 The difference of molecular alterations in NSCLC between eastern and southern cohorts

**Fig. 3:**
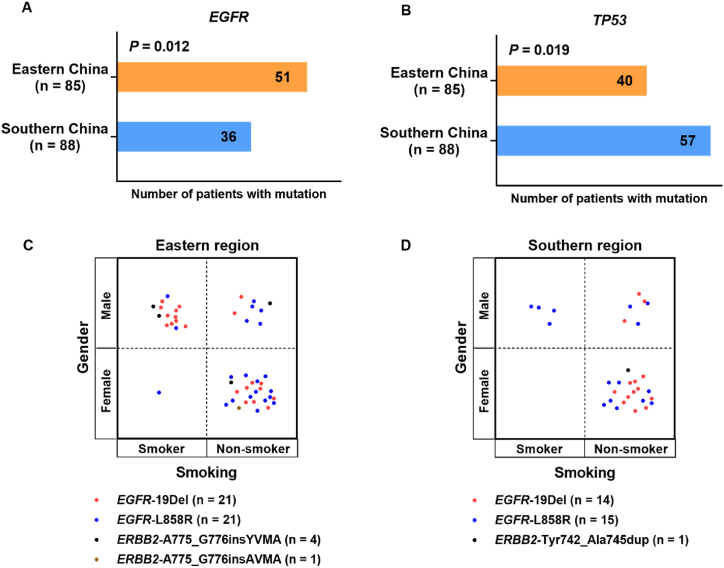
Comprehensive analysis of genetic mutations in eastern and southern NSCLC patients. **(A, B)** Comparison of number of patients with *EGFR* or *TP53* mutation between the two cohorts. **(C, D)** The clinical characteristics of patients with common driver mutations in eastern and southern cohorts.

The landscape of driver mutations in patients with NSCLC from Eastern (Fig. 1A) and Southern (Fig. 1B) regions displayed a region-specific mutational profile. Chi-square analyses were performed to investigate the difference in gene mutation frequency between eastern and southern cohorts (Supplementary Table 1). For instance, the prevalence of *EGFR* mutation was significantly higher in patients from eastern than that in those from southern China (60.0% *vs* 40.9%, *P* = 0.012; Fig. 3A). On the contrary, *TP53* mutated less in eastern patients compared with southern patients (47.1% *vs* 64.8%, *P* = 0.019; Fig. 3B). Furthermore, the mutation frequencies of *EGFR* 19Del were more common in female (*P* = 0.004) and nonsmokers (*P* < 0.001) in southern NSCLC, while the prevalence of *EGFR* L858R occurred more in female (*P* = 0.009) and nonsmokers (*P* < 0.001) in eastern NSCLC (Table 2). *ERBB2* exon 20 insertions (20ins) were identified in 6 patients, of which 4 patients from eastern region harboring A775_G776insYVMA, one patient from eastern region harboring A775_G776insAVMA, and one patient from southern region harboring Tyr742_Ala745dup (Fig. 3C and D).

**Table 2:** The distribution of *EGFR* hotspots in eastern and southern regions.

Characteristic	Eastern China		Southern China	
	*EGFR* L858R	*P*-value	*EGFR* 19Del	*P*-value
**Gender**		0.009		0.004
Male	7 (14.3%)		3 (6.0%)	
Female	14 (38.9%)		11 (28.9%)	
**Smoking history**		<0.001		<0.001
Yes	3 (7.5%)		0	
No	18 (40.0%)		14 (26.4%)	

### 3.4 Mutational signature for NSCLC according to the base substitutions

**Fig. 4:**
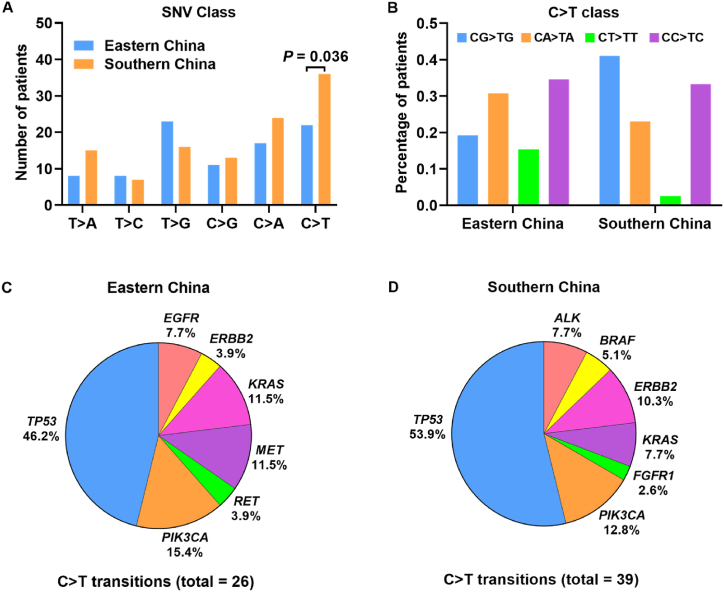
Base substitutions of SNVs in eastern and southern NSCLC. (**A**) Different class of the base substitutions of SNVs between eastern and southern patients. (**B**) Percentage of patients with C > T transitions localized in CG, CT, CA, and CC dinucleotides. (**C, D**) The prevalence of mutated genes with C > T transitions in eastern and southern patients.

We have found that the base substitutions of single-nucleotide variants were different between eastern and southern cohorts (Fig. 4A). The thymine to guanine (T > G) transversion was the most common base substitution type in eastern cohort, while the cytosine to thymine (C > T) transition was the most frequent in southern cohort. Mutations with C > T transition were identified much more in southern samples than that in eastern samples (*P* = 0.036). Nearly 19.2% and 41% of C > T mutations occurred in CpG dinucleotides in patients from eastern and southern regions, respectively (Fig. 4B). Amino acid changes caused by C > T substitutions were mainly related with arginine (Arg) (Table 3). Among all the mutated genes related with C > T transition, *TP53* mutated most frequently in either eastern or southern NSCLC patients (Fig. 4C and D).

**Table 3:** Distribution of gene mutations with C > T transitions occurred in the context of CpG dinucleotides.

Southern China				Eastern China			
Gene	Mutant	Codon	Cases	Gene	Mutant	Codon	Cases
*ALK*	p.Arg1231Gln	CGG > CAG	1	*EGFR*	p.Thr790Met	ACG > ATG	1
*ALK*	p.Arg1575His	CGT > CAT	1	*PIK3CA*	p.Val344Met	GTG > ATG	1
*ALK*	p.Arg291His	CGC > CAC	1	*TP53*	p.Arg156Cys	CGC > TGC	1
*FGFR1*	p.Gly849Arg	GGA > AGA	1	*TP53*	p.Arg248Trp	CGG > TGG	1
*PIK3CA*	p.Val344Met	GTG > ATG	1	*TP53*	p.Arg213Ter	CGA > TGA	1
*TP53*	p.Arg158His	CGC > CAC	1				
*TP53*	p.Arg175His	CGC > CAC	1				
*TP53*	p.Arg213Ter	CGA > TGA	1				
*TP53*	p.Arg248Gln	CGG > CAG	1				
*TP53*	p.Arg248Trp	CGG > TGG	2				
*TP53*	p.Arg273His	CGT > CAT	1				
*TP53*	p.Val31Ile	GTT > ATT	1				

## 4 Discussion

Genomic alterations of Chinese NSCLC patients were investigated in two independent cohorts. Similar to recent studies [[1], [2], [3], [4]], Chinese patients had a much higher frequency of *EGFR* mutations but a significantly lower frequency of *KRAS* mutation than Western populations. Additionally, higher rate of *EGFR* mutations was correlated with female and non-smokers, whereas the *KRAS* mutation was more common in male and patients with smoking history, which was in accordance with previous studies [12,13]. Moreover, *EGFR* mutation frequency was higher in early-stage patients, whereas *TP53* mutation had a correspondingly lower incidence, which may be due to the significant malignancy of *TP53* mutation leading to higher initial diagnosis stages.

To the best of our knowledge, somatic mutations of *EGFR* are key cancerous drivers in non-small-cell lung cancer (NSCLC), contributing to the vast majority of reported NSCLC cases in Asians [14,15]. However, limited studies have been reported about the *EGFR* mutation distribution across different regions of lung cancer patients. It was reported that uncommon or complex *EGFR* mutation rates were found to be higher in specimens from Xuanwei compared with other areas in China [5,16,17], however, the underlying molecular mechanism of this feature has not been fully understood yet. In our study, the frequency of *EGFR* mutations in eastern cohort was higher than that in southern cohort, indicating a region-related *EGFR* mutation frequency. This difference in *EGFR* incidence between the two cohorts might be due to the discrepancies in geographic location disparity or the corresponding variety in environmental characteristics. Smoking history [18,19], indoor cooking [20,21], air pollution exposure (e.g., PM2.5) [22,23], and exposure to toxic chemicals (e.g., radon, asbestos, and arsenic) [24,25] have been reported as key factors in the incidence of NSCLC. However, except for the smoking history discussed broadly in several research [[26], [27], [28]] and this study, other factors are challenging to quantify at the level of a certain individual, which made it difficult to accurately determine the correlation and potential mechanisms between geographic or environmental factors and certain types of gene mutations. In the future, larger cohorts and better consideration of patient information collection may make systematic analysis possible.

In the present study, the C > T mutations was the most frequent mutation type in the patients from southern China. Transition of C > T is one of the most common DNA modifications across somatic mutational signatures [29]. Most of these mutations are likely related to the relatively elevated rate of spontaneous deamination of 5-methyl-cytosine, which results in C > T transitions predominantly occur at CpG dinucleotides [30]. This also helps explain why so many mutations in this study alter arginine residues, as four of the six codons encoding arginine have CpG dinucleotides in the first two positions of their respective codons. Similar results have been described previously in *TP53* gene mutations [31]. In this study, the frequency of *TP53* mutation was significantly higher in NSCLC patients from southern region than that from eastern region. Studies reported that *TP53* was frequently mutated in nonmelanoma skin cancer, and C > T transitions had been observed as signature mutations after UV irradiation [32,33]. Southern China with more sufficient sunshine may indeed have more *TP53*-related mutations than eastern China. These findings therefore suggest that C > T mutagenesis likely to be a potential factor contributing to the pathogenesis and progression of lung cancer in southern cohort.

According to the National Comprehensive Cancer Network (NCCN) guidelines for NSCLC, biomarker tests for *EGFR* mutation, *ALK* rearrangement, *ROS1* rearrangement, *BRAF* V600E mutation, *NTRK1/2/3* gene fusion, *METex14* skipping mutation, *RET* rearrangement, and PD-L1 expression are recommended for all LUAD patients and partially recommended for LUSC patients. Incorporation of the above genetic tests could help to guide targeted therapies at the individual level. Given that all patients enrolled in our study were primarily diagnosed as NSCLC without any additional therapy, the positive results of *EGFR* mutation (87 patients), *ALK* rearrangement (8 patients), *ROS1* rearrangement (1 patient), *BRAF* V600E mutation (1 patient), and *METex14* skipping mutation (1 patient) have provided great guidance to help these patients with individually targeted medications.

It has been believed that both *EGFR* 19Del and L858R are sensitive to EGFR tyrosine kinase inhibitors (TKIs) [34]. Recent studies have demonstrated that patients with L858R showed significantly less response to TKIs than those with 19Del [35,36]. The distribution *of EGFR* 19Del and L858R were found different in NSCLC between the two cohorts, suggesting more accurate therapeutic approaches are necessary for patients from different regions. In the present study, it is worth noting that different *ERBB2* 20ins subtypes was identified in patients between the two regions. TKIs targeting *ERBB2* have limited activity in patients with *ERBB2*-positive tumors [37,38], although some case series have reported that lung cancer patients with *ERBB2* 20ins can benefit from TKIs, such as afatinib [39] and poziotinib [40]. However, response heterogeneity was observed in patients harboring different 20ins subtypes [39,41]. Thus, further efforts are needed to understand the underlying mechanism, and additional clinical validations in larger cohorts will be necessary to confirm our findings.

NGS technology is widely applied in clinical practice for monitoring genetic landscape of different types of tumors [42,43]. For instance, detection of several driver mutations is recommended for improving personal medication in NSCLC patients. The advantages of NGS include the ability to sequence a large panel of genes at the same time, the high sensitivity of rare mutations, and a relatively low cost [44]. However, the balance between sequencing panel size, sequencing depth, and cost must be considered in clinical applications. Moreover, NGS has potential deficiencies in the clinical sensitivity of detecting technically challenging variants (e.g., large indels, small CMVs, and variants in highly homologous regions) [[45], [46], [47]]. As investigated in a recent interlaboratory pilot study, it is temporarily impossible to capture all challenging variant types in a single NGS platform or variant calling algorithm [48]. For the benefit of our research, the engagement of larger NGS panels and improvement of clinical-related challenging variants identification may further clearly state the distinction between genetic alteration characteristics of different cohorts.

Finally, it is essential to discuss the existing limitations of this study. The sample size of our cohorts was relatively small, which may impact the generalizability of the findings. Moreover, we recruited over 90% of LUAD patients and fewer other types of NSCLC patients. However, accounting for the different genetic characteristics between LUAD and LUSC, the conclusions drawn in this article may be more applicable to LUAD patients. In the future, larger cohorts and an average enrollment of participants would help us to validate the current findings and better explore the mutational profile of NSCLC in the eastern and southern Chinese cohorts. Moreover, our research was initially limited to exploring the genetic alterations in primary NSCLC patients between different cohorts, and further analysis of healthy controls or metastatic tumors would enable us to better understand the tumor-specific or metastatic-associated gene mutation in larger cohorts.

## 5 Conclusions

The differences in the mutational features of NSCLC patients between eastern and southern China have been revealed. Results of eastern cohort showed higher frequency of *EGFR* mutations, lower prevalence of *TP53* mutations and fewer C > T transitions compared with those in the southern patients. These distinct patterns of mutation could provide clues for the mechanism of carcinogenesis of lung cancer, offering opportunities to stratify patients into optimal treatment plans based on genomic profiles.

## Funding sources

This work was supported by the Shanghai Municipal Health Commission Clinical Research Project (No. 202240394).

## Ethical statement

The authors are accountable for all aspects of the work in ensuring that questions related to the accuracy or integrity of any part of the work are appropriately investigated and resolved. The study was conducted in accordance with the Declaration of Helsinki (as revised in 2013). Written informed consent was obtained from all patients before genetic analysis of biological samples. The study was approved by the Ethics Committee of Shanghai Changzheng Hospital (2019SL009) and Central Hospital of Guangdong Nongken (No.2018001), separately.

## Author contribution statement

Chengdong Liu: Conceived and designed the experiments; Wrote the paper.

Kangbao Li: Conceived and designed the experiments.

Yi Sui: Analyzed and interpreted the data; Wrote the paper.

Hongmei Liu, Tianmin Xiang: Analyzed and interpreted the data.

Yunzhi Zhang, Yuan Lu: Contributed reagents, materials, analysis tools or data.

Wei Lu, Suqian Xu, Yongfeng Chen: Performed the experiments.

Gehui Wang: Analyzed and interpreted the data; Contributed reagents, materials, analysis tools or data.

Yongguang Cai, Kenan Huang: Conceived and designed the experiments; Wrote the paper.

## Data availability statement

Data included in article/supp. Material/referenced in article.

## Declaration of competing interest

The authors declare the following financial interests/personal relationships which may be considered as potential competing interests:

YS, HL, YZ, GW, SX and TX were employed by company Singlera Genomics (Shanghai). The remaining authors declare that the research was conducted in the absence of any commercial or financial relationships that could be construed as a potential conflict of interest.
