# Supplementary material for: Different gene alterations in patients with non-small-cell lung cancer between the eastern and southern China
Source: Heliyon. 2023 Sep 14;9(10):e20171. doi: 10.1016/j.heliyon.2023.e20171 (PMC10520317; doi:10.1016/j.heliyon.2023.e20171)
Supplement: Multimedia component 1 [file mmc1.docx]

**Different Gene Alterations in Patients with Non-Small-Cell Lung Cancer between the eastern and southern China**

Chengdong Liu^a,1^_,_ Kangbao Li^b,1^, Yi Sui^c^, Hongmei Liu^c^, Yunzhi Zhang^c^, Yuan Lu^d^, Wei Lu^d^, Yongfeng Chen^d^, Gehui Wang^c^, Suqian Xu^c^, Tianmin Xiang^c^, Yongguang Cai^d,*^ and Kenan Huang^e,^*

^a^ Department of thoracic surgery, Naval Medical Center of PLA, 338 Huaihai Road, Changning District, Shanghai 200052, China

^b^ Department of Geriatrics, Gastroenterology Ward, Guangzhou First People’s Hospital, School of Medicine, South China University of Technology, Guangzhou 510180, China

^c^ Singlera Genomics Inc., Shanghai 201318, China

^d^ Medical Oncology Department V, Central Hospital of Guangdong Nongken 524002, China

^e^ Department of Thoracic Surgery, Shanghai Changzheng Hospital, Navy Military Medical University, 415 Fengyang Road, Huangpu District, Shanghai 200003, China

^1^ The author had the same contribution to this work.

**Supplementary Table 1. Chi-square test between genes mutation status and patients with different gender, smoking history, stage, and cohort.**

| **Genes** | **Gender** | |  | **Smoking history** | |  | **Stage** | |  | **Cohort** | |
| --- | --- | --- | --- | --- | --- | --- | --- | --- | --- | --- | --- |
|  | χ^2^ value | *P*-value |  | χ^2^ value | *P*-value |  | χ^2^ value | *P*-value |  | χ^2^ value | *P*-value |
| *ALK* | 1.731 | 0.240 |  | 1.376 | 0.405 |  | 1.941 | 0.555 |  | 2.621 | 0.211 |
| *BRAF* | 0.251 | 0.617 |  | 0.636 | 0.425 |  | 4.547 | 0.272 |  | 2.246 | 0.134 |
| *EGFR* | 9.046 | 0.003* |  | 12.926 | <0.001* |  | 14.235 | 0.003* |  | 6.304 | 0.012* |
| *ERBB2* | 0.251 | 0.617 |  | 0.636 | 0.425 |  | 0.444 | 0.964 |  | 0.138 | 0.711 |
| *FGFR1* | 1.512 | 0.508 |  | 1.549 | 0.506 |  | 0.807 | 1.000 |  | 0.001 | 1.000 |
| *KRAS* | 10.434 | 0.001* |  | 14.554 | <0.001* |  | 2.797 | 0.466 |  | 1.509 | 0.219 |
| *MET* | 0.576 | 0.448 |  | 2.949 | 0.086 |  | 4.115 | 0.342 |  | 0.064 | 0.801 |
| *PIK3CA* | 0.796 | 0.372 |  | 0.036 | 0.849 |  | 2.221 | 0.654 |  | 0.032 | 0.857 |
| *RET* | 0.111 | 1.000 |  | 0.125 | 1.000 |  | 4.902 | 0.128 |  | 3.161 | 0.116 |
| *TP53* | 0.213 | 0.644 |  | 0.830 | 0.362 |  | 15.166 | 0.002* |  | 5.508 | 0.019* |

*There was a significant difference.
